# Promotion of Healthy Lifestyles Alone Might Not Substantially Reduce Socioeconomic Inequity-Related Mortality Risk in Older People in China: A Prospective Cohort Study

**DOI:** 10.1007/s44197-023-00095-3

**Published:** 2023-03-04

**Authors:** Ziqiong Wang, Yi Zheng, Haiyan Ruan, Liying Li, Sen He

**Affiliations:** 1 Department of Cardiology, West China Hospital of Sichuan University, No. 37 Guoxue Alley, Chengdu, China; 2 Department of Cardiology, Hospital of Traditional Chinese Medicine, Shuangliu District, Chengdu, China

**Keywords:** All-cause mortality, China, Healthy lifestyle, Older people, Socioeconomic status

## Abstract

**Background:**

Whether healthy lifestyles mediate the association of socioeconomic status (SES) with mortality in older people is largely unknown.

**Methods:**

A total of 22,093 older participants (age ≥ 65 years) from 5 waves (2002–2014) of Chinese Longitudinal Healthy Longevity Survey cohort were included for analysis. Mediation analysis of lifestyles on the association of SES with all-cause mortality was conducted.

**Results:**

During a mean follow-up period of 4.92 ± 4.03 years, 15,721 (71.76%) deaths occurred. Compared with high SES, medium SES increased the risk of mortality by 13.5% (HR [total effect]: 1.135, 95% CI 1.067–1.205, *p* < 0.001), and the total effect was not mediated by healthy lifestyles (mediation proportion: − 0.1%, 95% CI − 3.8 to 3.3%, *p* = 0.936). The total effect when participants of low SES were compared with participants of high SES was HR = 1.161 (95% CI 1.088–1.229, *p* < 0.001) for mortality, and the total effect was modestly mediated through healthy lifestyles (mediation proportion: − 8.9%, 95% CI − 16.6 to − 5.1%, *p* < 0.001). Stratification analyses by sex, age and comorbidities, as well as a series of sensitivity analyses indicated similar results. In addition, mortality risk showed a downward trend with increased number of healthy lifestyles within each SES level (all *p* for trend < 0.050).

**Conclusion:**

Promotion of healthy lifestyles alone can only reduce a small proportion of socioeconomic inequity-related mortality risk in older Chinese people. Even so, healthy lifestyles are important in reducing the overall mortality risk within each SES level.

**Supplementary Information:**

The online version contains supplementary material available at 10.1007/s44197-023-00095-3.

## Background

Socioeconomic status (SES) is defined as a measure of individual’s combined economic and social status, which describes the position an individual occupies in the structure of society, and generally reflected from multiple dimensions, such as education, occupation, and income [1–3]. Plenty of epidemiological studies have widely reported the inverse association between SES and health. Socioeconomically disadvantaged individuals were at higher risk of non-communicable diseases and mortality [4–8]. Lifestyles and mortality are related [9, 10], and lifestyle factors are often regarded as important factors mediating the relationship between SES and mortality. However, the results are inconsistent. Méjean et al. demonstrated that diet and lifestyle (mainly alcohol consumption and smoking) explained over 70% of the educational differences in coronary artery disease and stroke and 65% of employment status-related differences in coronary heart disease in EPIC-NL cohort [11]. Stringhini et al. reported that healthy behaviors attenuated the association of SES with mortality by 75% in Whitehall II cohort and 19% GAZEL cohort [12]. While Zhang et al. demonstrated that healthy lifestyles only improved a small proportion (3–12%) of the socioeconomic inequity-related mortality and cardiovascular diseases in U.S. and U.K. adults in US NHANES and UK biobank cohorts [13]. Therefore, to what extent lifestyle mediates the association between SES and ill health remains debatable. Furthermore, those studies mentioned before mainly focused on the general population or middle-aged population [11–13], the data about mediated role of lifestyles in socioeconomic inequity-related ill health for older people are extremely limited. Facing the population aging throughout the world, and also the fact that the lion’s share of mortality occurs at old age and the simultaneously increased life expectancy, SES-related health inequities in older adults are a critical issue in the world, including China.

China has entered moderately aging society by the end of 2021, and the population aged 65 and above reached 200 million, accounting for more than 14.2% of the whole population [14]. Most of them were born and received education in 1920s–1940s. According to the report of People’s Daily China (China state-affiliated media), more than 80% of the Chinese population were illiterate at early stage of the new China [15], which indicated that the educational level was severely lagging behind prior to the foundation of People’s Republic of China in 1949. Furthermore, considering the limited socioeconomic development back then, a vast majority of Chinese people were hence characterized with low SES. In the subsequent decades after the foundation of new China, the government has made its efforts to increase literacy and to provide education [16], as well as provide more diversified occupations, such as industry, commerce, construction, scientific research, public health, government agencies and people’s organizations [17]. From the late 1980s, society and economy have further developed rapidly since the start of market-oriented reform. However, following the policy of “let some people get rich first”, the improvement was not distributed uniformly over different regions [18]. Given the unchangeable nature of education and occupation but the variable income during the lifetime of these older people, their SES has inevitably widened. At the same time, socioeconomic inequities in health status also became prominent among population groups in China [19].

The blueprint of Healthy China 2030 set out the goal of effectively controlling major health risk factors in 2030, including different lifestyles, such as cigarette smoking, alcohol consumption, inactivity, and unhealthy diet. Therefore, in the present study, we aimed to investigate whether healthy lifestyles could mediate the association of SES with mortality in older Chinese people using the data from the Chinese Longitudinal Healthy Longevity Survey (CLHLS) cohort, and also to examine the role of healthy lifestyles within different levels of SES.

## Methods

### Study Participants

**Fig. 1:**
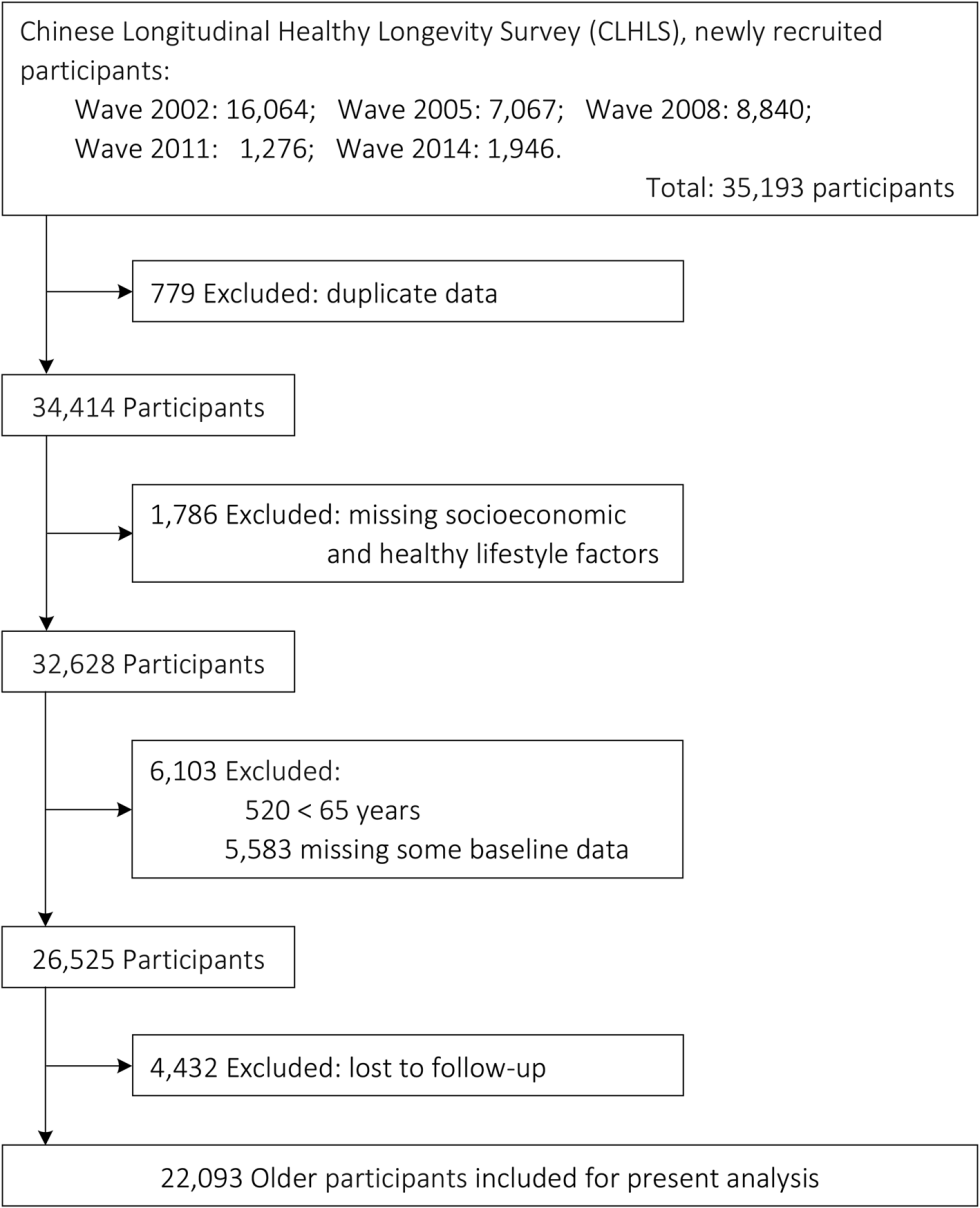
Study flow diagram

The CLHLS is a nationwide, ongoing, prospective cohort study of community-dwelling Chinese older people. It began in 1998, with subsequent follow-ups in 2000, 2002, 2005, 2008, 2011, 2014, and 2018. To reduce the attrition due to death and loss to follow-up, new participants are enrolled during the following waves from 1998. The CLHLS is conducted in a randomly selected half of the counties and cities in 23 of the 31 provinces, covering about 85.0% of Chinese population, and administered in participants’ homes by trained interviewers. More details about CLHLS have been reported elsewhere, and data quality was reported to be generally good [20, 21]. Since income, an important socioeconomic factor, was collected since wave 2002, the present study included participants from wave 2002 to wave 2014 (total of 5 waves), and these participants were followed up until 2018. According to the inclusion and exclusion criteria, 22,093 older participants (age ≥ 65 years) were finally included for the present analysis (Fig. 1). Spatial distributions of the study population are shown in eFig. 1.

Research Ethics Committee of Peking University approved the study (IRB00001052-13074), which was performed in accordance with the principles of the Declaration of Helsinki. The survey respondents gave informed consent before participation.

### Assessment of Socioeconomic Status

According to previous studies [2, 22], we used education level, occupation, and self-reported family income level to measure SES, and each factor was divided into three levels (low, medium, and high) with consideration of practical interpretation and sample size within levels. Based on the question “How many years did you attend school?”, education level was categorized into no school, primary school, and middle school or more. All participants in our study have retired, and we then grouped participants according to primary occupation before age 60: low occupational grade, medium occupational grade, and high occupational grade [23]. Based on the question “How do you rate your economic status compared with others in your local area?”, the income was bracketed into three categories: poor, fair, and rich. More details about socioeconomic factors were in eMethods.

For each socioeconomic factor, we assigned 1 point for a low level, 2 points for a medium level, and 3 points for a high level. The SES score assigned to each participant was the sum of education level, occupation, and income scores, as previously described [23, 24]. For ease of presentation and interpretation, we performed statistical analyses after stratifying by the range and distribution of SES score (eFig. 2): low SES (score = 3, and 4), medium SES (score = 5, and 6), and high SES (score = 7, 8, and 9).

### Assessment of Healthy Lifestyles

For assessing healthy lifestyles, we constructed a healthy lifestyle score including cigarette smoking, alcohol consumption, physical activity, and diet according to a previous study [13]. In the present study, we defined never smoking as a healthy level. Chinese dietary guidelines (2018) [25]recommends that adults should drink no more than 15 g of pure alcohol per day; therefore, we defined a healthy level as never drinking or daily consumption of ≤ 15 g pure alcohol in the past or at present. i.e., no heavy alcohol consumption. WHO 2020 guidelines on physical activity and sedentary behavior defines physical activity as any bodily movement produced by skeletal muscles that requires energy expenditure [26]; therefore, participants who exercised regularly at present or participated in any active activity (e.g., raising domestic animals, doing house work) almost every day were defined as a healthy level in our study. According to a previous CLHLS study [27], we calculated healthful diet index, and the top three quintiles of healthful diet index was defined as a healthy diet. eMethods shows more detailed information of healthy lifestyle factors.

For each lifestyle factor, we assigned 1 point for a healthy level and 0 point for an unhealthy level. Thus, the healthy lifestyle score was the sum of the points and ranged from 0 to 4, with higher scores indicating healthier lifestyles, and the method has been used widely [23, 24].

### Assessment of Other Covariates

eTable 1 shows the detailed information of other covariates, which were obtained through questionnaires, including: sex, age, education, marital status, residence, co-residence, comorbidities (hypertension, diabetes, heart diseases, cerebrovascular diseases, respiratory diseases, and cancer), activities of daily living (ADL) disability, and self-reported health. More detailed information about these covariates can be found on: https://agingcenter.duke.edu/CLHLS.

### Study Outcome

The study outcome was all-cause mortality (referred as mortality later), and the survival status and date of death were collected through the deceased participant’s close family members during each survey. All participants were followed from the first evaluation up to death or the most recent evaluation. In the CLHLS, the reliability of mortality may be more reliable than those obtained from the national census, although some recall errors occurred [28].

### Statistical Analysis

Baseline characteristics were described across different levels of SES. Continuous variables were tested for normality using the Shapiro–Wilk test and reported as mean and standard deviation when normally distributed and as median (inter-quartile range, IQR) otherwise. For continuous variables, *p* value for trend was computed from the Pearson test when row-variable was normal distribution and from the Spearman test when it was non-normal distribution. When the row-variable was categorical, *p* value for trend was computed from Mantel–Haenszel test of trend. Cox proportional hazards regression models were used to evaluate the independent association of SES and healthy lifestyle score with mortality. The proportional hazards assumption was examined by calculating the beta coefficients of SES and healthy lifestyle score for mortality over time, which ascertained the assumption was not violated.

**Fig. 2:**
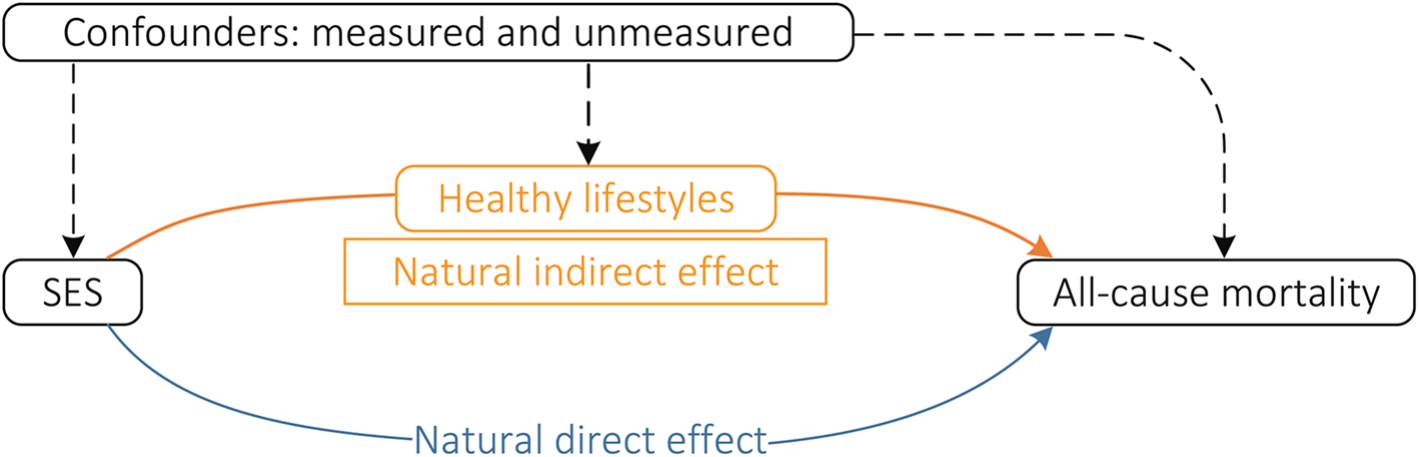
Directed acyclic graph depicting the hypothesized associations between exposure SES, mediator healthy lifestyles, outcome all-cause mortality, and the measured and unmeasured confounders. Natural direct (blue solid line) and natural indirect (red–orange solid line) effect estimated the effects of SES on all-cause mortality that does not or does act through the mediator (i.e., healthy lifestyles), respectively. All statistical models were based on this structure and were adjusted for potential confounders (dashed arrows), including sex, age, marital status, residence, co-residence, comorbidities, ADL disability, and self-reported health. In addition, the possibility of unmeasured confounding, which can never be ruled out in observational research, is also indicated with dashed arrows. *SES* socioeconomic status

Using the counterfactual framework [29, 30], we applied a new 2-stage regression method [29, 30] for causal mediation analysis to estimate the direct and indirect effects using the R package (CMAverse) [31]. In brief, two regression models were fitted by the data: one modeling the outcome (i.e., mortality, Cox proportional hazards regression) and the other modeling the mediator (i.e., healthy lifestyle score, linear regression). In the two models, we used SES as categorical variable (low, medium, and high), and healthy lifestyle score was used as a linear term because a Cox proportional hazards regression model using the score as categorical variable confirmed a linear association between the score and the risk of mortality (*p* for trend < 0.001). All models were adjusted for potential confounders. Standard errors of effects was assessed through bootstrapping method (1000 bootstrapped samples) [31]. Assuming associations between variables as shown in the directed acyclic graph in the Fig. 2, and total causal effect of SES on mortality (expressed as the hazard ratio [HR] vs. the reference high SES) could be decomposed into two components: natural direct effect (i.e., the effect of SES not explained through the mediator), and natural indirect effect (i.e., the effect of SES that was due to the mediator) [29, 30]. Meanwhile, the proportion of the association between SES and mortality mediated through healthy lifestyles was calculated on the log-transformed HR scale as log(indirect effect HR)/log(total effect HR), since HRs are additive on this scale. In addition, it is possible that the indirect effect takes different values depending on the baseline exposure status, and we tested the statistical significance of interaction between SES and healthy lifestyles.

To assess the robustness of main results, as well as potential variations in different subgroups, we performed a series of stratification and sensitivity analyses, including: (1) we evaluated total, direct and indirect effects by sex, and also assessed these effects by age. We also examined these effects in participants with or without comorbidities because both SES and lifestyles could be influenced by major chronic diseases; (2) to exclude the deaths that occurred within the first year of follow-up to reduce potential reverse causation; (3) to address the issue of loss to follow-up, we conducted another sensitivity analysis by considering the losses censored at two time points: median and the end of follow-up; (4) to mitigate potential bias caused by missing data, we performed mediation analysis after multiple imputation.

Finally, we further examined the independent association of healthy lifestyles with mortality within different levels of SES (low, medium, and high), and assessed the interaction of SES and healthy lifestyles with mortality. Multiple sensitivity analyses were also performed to evaluate the robustness of the findings.

All analyses were performed with R version 4.1.0, mainly including the “compareGroups”, “CMAverse”, “tidyverse”, “rms”, “mice” and “stats” packages (http://www.R-project.org). All tests were two sided, and *p* values < 0.05 were considered statistically significant.

## Results

### Baseline Characteristics

Our study included 22,093 older participants. The median age of the study population was 88.00 (IQR 77.00, 96.00) years, and 9,511 (43.05%) of the participants were men. Table 1 shows baseline characteristics presented by the levels of SES. Participants of low SES tended to be less educated, and low occupational grade before age 60, and to have low income. Never smoking, no heavy alcohol consumption, and healthy diet were more prevalent among participants of low SES; conversely, regular physical activity was more prevalent among participants of high SES. In addition, participants of low SES were more likely to be women, older, not in marriage, living in rural areas, and living alone, and to have lower proportion of comorbidities, and greater prevalence of ADL disability and poor self-reported health. eTable 2 shows baseline characteristics of participants included or excluded from the current analysis owing to missing information.

**Table 1:** Baseline characteristics of the study participants by different levels of socioeconomic status

	All (*n* = 22,093)	Low SES (*n* = 11,600)	Medium SES (*n* = 8110)	High SES (*n* = 2383)	*p* for trend
Sex: male	9511 (43.05%)	3026 (26.09%)	4586 (56.55%)	1899 (79.69%)	< 0.001
Age (years)	88.00 (77.00, 96.00)	91.00 (81.00, 100.00)	86.00 (74.00, 94.00)	80.00 (70.00, 90.00)	< 0.001
Marital status					< 0.001
In marriage	7291 (33.00%)	2676 (23.07%)	3197 (39.42%)	1418 (59.50%)	
Not in marriage	14,802 (67.00%)	8924 (76.93%)	4913 (60.58%)	965 (40.50%)	
Residence					< 0.001
Urban	8851 (40.06%)	3472 (29.93%)	3648 (44.98%)	1731 (72.64%)	
Rural	13,242 (59.94%)	8128 (70.07%)	4462 (55.02%)	652 (27.36%)	
Co-residence					< 0.001
With family members	18,292 (82.80%)	9323 (80.37%)	6880 (84.83%)	2089 (87.66%)	
Alone	3213 (14.54%)	1983 (17.09%)	1028 (12.68%)	202 (8.48%)	
In an institution	588 (2.66%)	294 (2.53%)	202 (2.49%)	92 (3.86%)	
Comorbidities					
Hypertension	3756 (17.00%)	1785 (15.39%)	1397 (17.23%)	574 (24.09%)	< 0.001
Diabetes	476 (2.15%)	147 (1.27%)	204 (2.52%)	125 (5.25%)	< 0.001
Heart diseases	1787 (8.09%)	730 (6.29%)	675 (8.32%)	382 (16.03%)	< 0.001
Cerebrovascular diseases	1070 (4.84%)	488 (4.21%)	392 (4.83%)	190 (7.97%)	< 0.001
Respiratory diseases	2524 (11.42%)	1243 (10.72%)	947 (11.68%)	334 (14.02%)	< 0.001
Cancer	92 (0.42%)	40 (0.34%)	34 (0.42%)	18 (0.76%)	0.012
ADL disability	5145 (23.29%)	3072 (26.48%)	1678 (20.69%)	395 (16.58%)	< 0.001
Self-reported health					< 0.001
Good	11,281 (51.06%)	5319 (45.85%)	4552 (56.13%)	1410 (59.17%)	
Fair	7522 (34.05%)	4123 (35.54%)	2642 (32.58%)	757 (31.77%)	
Poor	3290 (14.89%)	2158 (18.60%)	916 (11.29%)	216 (9.06%)	
Education					< 0.001
Middle school or more	2040 (9.23%)	0 (0.00%)	493 (6.08%)	1547 (64.92%)	
Primary school	6129 (27.74%)	585 (5.04%)	4739 (58.43%)	805 (33.78%)	
No school	13,924 (63.02%)	11,015 (94.96%)	2878 (35.49%)	31 (1.30%)	
Occupation before 60 years					< 0.001
High occupational grade	1602 (7.25%)	0 (0.00%)	116 (1.43%)	1486 (62.36%)	
Medium occupational grade	3432 (15.53%)	185 (1.59%)	2445 (30.15%)	802 (33.66%)	
Low occupational grade	17,059 (77.21%)	11,415 (98.41%)	5549 (68.42%)	95 (3.99%)	
Income					< 0.001
Rich	3676 (16.64%)	0 (0.00%)	2499 (30.81%)	1177 (49.39%)	
Fair	14,959 (67.71%)	8482 (73.12%)	5311 (65.49%)	1166 (48.93%)	
Poor	3458 (15.65%)	3118 (26.88%)	300 (3.70%)	40 (1.68%)	
Never smoking	14,769 (66.85%)	8844 (76.24%)	4730 (58.32%)	1195 (50.15%)	< 0.001
No heavy alcohol consumption	16,205 (73.35%)	9160 (78.97%)	5478 (67.55%)	1567 (65.76%)	< 0.001
Regular physical activity	14,823 (67.09%)	7111 (61.30%)	5775 (71.21%)	1937 (81.28%)	< 0.001
Healthy diet	13,587 (61.50%)	8222 (70.88%)	4441 (54.76%)	924 (38.77%)	< 0.001

Values are median (IQR) or *n* (%)

*ADL* activities of daily living, *IQR* inter-quartile range, *SES* socioeconomic status

### Mediation Analysis of Lifestyle on the Association of SES with Mortality

During a mean follow-up period of 4.92 ± 4.03 years, 15,721 (71.76%) deaths were recorded. In Cox proportional hazards models adjusted for potential confounders, lower SES score was associated with increased risk of mortality, and high healthy lifestyle score was associated with reduced risk of mortality (eTables 3 and 4). All socioeconomic and healthy lifestyle factors were also associated with the risk of mortality, except for income and healthy diet (eTables 3 and 4).

Table 2 shows the results of mediation analysis. Compared with high SES, medium SES increased the risk of mortality by 13.5% (HR [total effect]: 1.135, 95% CI 1.067–1.205, *p* < 0.001), and the total effect was not mediated by healthy lifestyles (mediation proportion: − 0.1%, 95% CI − 3.8 to 3.3%, *p* = 0.936) (Table 2). The total effect when participants of low SES were compared with participants of high SES was HR = 1.161 (95% CI 1.088–1.229, *p* < 0.001) for mortality, and the total effect was partially mediated through healthy lifestyles (mediation proportion: − 8.9%, 95% CI − 16.6 to − 5.1%, *p* < 0.001) (Table 2). In addition, the interaction between the exposure (i.e., SES) and the mediation (i.e., healthy lifestyles) was not statistically significant, and the excess relative HRs solely due to interaction were − 0.0021 (95% CI − 0.0960 to 0.1300, *p* = 0.932) for medium SES, and − 0.0363 (95% CI − 0.1313 to 0.0590, *p* = 0.438) for low SES, respectively (Table 2).

**Table 2:** Mediation (by healthy lifestyles) analysis of socioeconomic status and all-cause mortality

	High SES	Medium SES	Low SES
No. of participants (*n*)	2383	8110	11,600
Deaths (*n*)	1298	5514	8909
Follow-up (PYs)	13,608.3	42,001.4	53,066.1
Mortality rate (95% CI)^a^	9.5 (9.0–10.0)	13.1 (12.8 to 13.5)	16.8 (16.5 to 17.1)
Association^b^			
Total effect; HR (95% CI), *p*	1 [Reference]	1.135 (1.067 to 1.204), < 0.001	1.161 (1.088 to 1.229), < 0.001
Natural direct effect; HR (95% CI), *p*		1.135 (1.067 to 1.205), < 0.001	1.175 (1.102 to 1.245), < 0.001
Natural indirect effect; HR (95% CI), *p*		1.000 (0.996 to 1.004), 0.936	0.988 (0.983 to 0.992), < 0.001
Mediation proportion; % (95% CI), *p*		− 0.1 (− 3.8 to 3.3), 0.936	− 8.9 (− 16.6 to − 5.1), < 0.001
Excess relative HR solely due to interaction^c^; HR (95% CI), *p*		− 0.0021 (− 0.0960 to 0.1300), 0.932	− 0.0363 (− 0.1313 to 0.0590), 0.438

All models were adjusted for sex, age, marital status, residence, co-residence, comorbidities, ADL disability, and self-reported health

*ADL* activities of daily living, *CI* confidence interval, *HR* hazard ratio, *PYs* person-years, *SES* socioeconomic status

^a^Per 100 PYs

^b^Natural direct effect and natural indirect effect estimated the effects of SES on mortality that did not or did act through the mediator (i.e., healthy lifestyles), respectively. Mediation proportion estimated the percent of SES effect, on the log(HR) scale, that acted through the mediator, i.e., healthy lifestyles. The results were calculated without considering exposure-mediator interaction

^c^The results were considered with exposure–mediator interaction. Since the interaction was not statistically significant, we only showed the excess relative HR that was solely due to interaction

### Stratification and Sensitivity Analysis

We performed a series of stratification and sensitivity analyses about mediating effects. Overall, these findings were largely consistent with the main results. In the subgroups by sex, age and comorbidities, results of all analyses did not change substantially, except that there was no mediating effect in the subgroup with age ≥ 88 years (eTable 5). Then, consistent results were observed after excluding participants who died within the first year of follow-up (eTable 6). Moreover, the results did not substantially change when we treated participants lost to follow-up as censored at the median or the end of follow-up (eTable 6). Finally, similar results were also observed after multiple imputation (eTable 6).

### Additional Analysis: Association of Healthy Lifestyles with Mortality Within Different Levels of SES

**Fig. 3:**
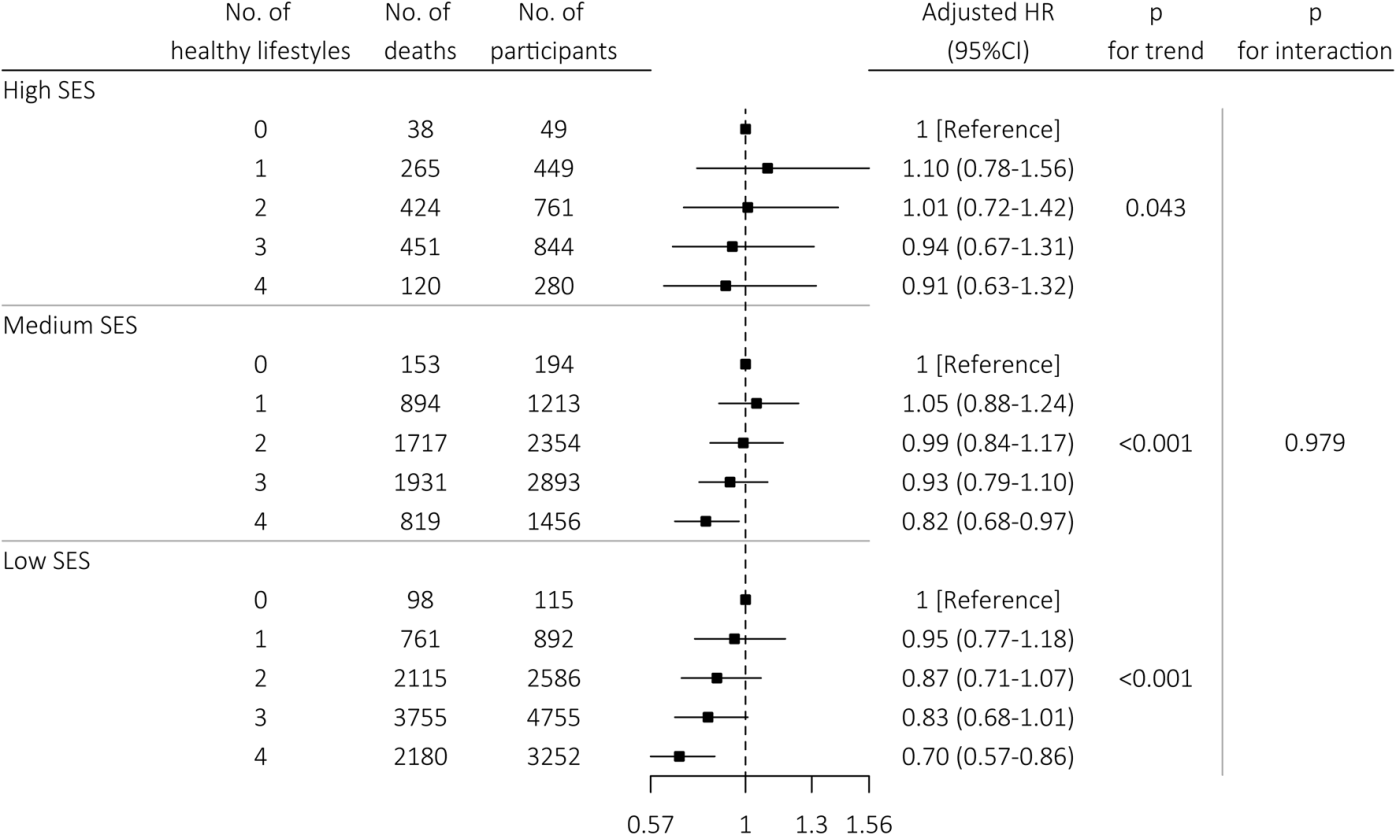
Association of healthy lifestyles with all-cause mortality at different levels of SES. ^a^The values obtained from Wald tests of a linear association of the score as a numeral (0–4) with the risk of all-cause mortality. All models were adjusted for sex, age, marital status, residence, co-residence, comorbidities, ADL disability, and self-reported health. *CI* confidence interval, *HR* hazard ratio, *SES* socioeconomic status

The above analysis suggested that healthy lifestyles might only reduce a small proportion of socioeconomic inequity-related mortality risk in the group of low SES, and could not reduce the socioeconomic inequity-related mortality risk in the group of medium SES. Therefore, we further examined the value of healthy lifestyles per se in reducing mortality risk within different levels of SES (low, medium, and high). Overall, mortality risk reduced with increased healthy lifestyle score in all levels of SES, and HRs were 0.94 (per one score increment, 95% CI 0.89–1.00, *p* = 0.043) for high SES group, 0.93 (95% CI 0.91–0.96, *p* < 0.001) for medium SES group, and 0.91 (95% CI 0.89–0.93, *p* < 0.001) for low SES group, respectively. When healthy lifestyle score was considered as categorical variable and compared zero healthy lifestyle, mortality risk was on a downward trend with increased number of healthy lifestyles within each level of SES (all *p* for trend < 0.050), especially in low and medium SES (Fig. 3), and sensitivity analyses also provided similar results (eTables 7, 8, 9).

## Discussion

In our CLHLS cohort, consistent with previous studies, an inverse association between SES and mortality was observed. When compared with high SES, medium and low SES significantly increased the risk of mortality by 13.5% and 16.1%, respectively. Healthy lifestyles attenuated the association of SES with mortality by 8.9% when low SES was compared with high SES but showed no impact when medium SES was compared with high SES, which indicated that healthy lifestyles only mediated a small proportion of the socioeconomic inequity-related mortality in older people of low SES. Stratification analysis by sex, age and comorbidities showed similar results, except for the extremely older people with age over 88 years old. Within different levels of SES, mortality risk was on a downward trend with increased number of healthy lifestyles, especially in low and medium SES, which indicated the importance of healthy lifestyles in reducing the overall mortality risk for older people.

In the present study, we observed significant mortality inequity by SES for older people, which were in line with these of past research [32–36]. To name a few examples, Bassuk et al. demonstrated that higher SES, as measured by education, by household income, or by occupational prestige, was generally associated with lower mortality in four American community-dwelling populations aged 65 years or more [32]. Using the data from the 2014 to 2015 Medicare Health Outcomes Survey, Kim revealed that mortality rates were significantly higher in low-income elders or non-homeowners when compared to their respective opposites [33]. Similarly, in another study consisted of 11 European populations, Huisman et al. found persisted socioeconomic inequity in mortality among older men and women, and the SES in this study was indicated by educational level or housing tenure [34]. However, these studies relied on single variable to determine the individual SES, which only reflected a limited proportion of overall SES. In our study, we used a comprehensive SES score compromising of family income, education level and occupation before their sixties to assess individual SES, which should be more convincible to draw the current conclusions.

Healthy lifestyles have been frequently proposed as factors mediating socioeconomic inequity in health. However, the quantification of the contribution of healthy behaviors to the socioeconomic gradient in health varied a lot. According to a previous meta-analysis, the association of lower SES and all-cause mortality and cardiometabolic disorders could be explained by healthy behaviors with a minimum contribution of − 43% and maximum contribution of 261% [37]. The huge differences may be explained by the heterogeneous nature of these studies. First, the methods to assess SES and healthy behaviors did not reach a consensus. For SES, single indicator, such as education, income, occupation and wealth, or combination of 2 ~ 3 indicators were used in different epidemiological studies. For healthy behaviors, the most commonly used four variables were smoking, alcohol, diet and physical activity, with single variable or combination of 2 ~ 4 variables used in different epidemiological studies. Second, participants in these studies were from different countries with different ethnicity backgrounds. Third, the level of social development was not synchronized during the survey period. Fourth, when looking at the age spectrum of those cohorts, they generally focused on the natural populations or middle-aged populations. The older people had been largely neglected. As we mentioned before, the participants in our study were born in 1920s–1940s and experienced the huge socioeconomic transformation during their lifetimes, resulting in the widened SES, as well as more pronounced socioeconomic inequity in health among different sub-populations. However, the socioeconomic inequity can be offset by lifestyle factors at only a small proportion in the older Chinese people. Other mechanisms of socioeconomic inequity in health for older people should be explored.

Within different levels of SES, healthy behaviors showed more protective effects in reducing the morality risk in participants with lower SES in our study. In another study conducted by Foster et al. analyzing the data from UK Biobank cohort, they also found that the poor lifestyle score was associated with more risk of all-cause mortality and cardiovascular mortality for individuals in the most deprived group than that of the most affluent socioeconomic group [38]. These findings echoed with the vulnerability hypothesis, which suggested that the same unhealthy lifestyle factors were more harmful for people with low SES than people with high SES [39]. Therefore, the promotion of healthy behaviors in lower SES people should be more encouraged. Especially, in some developing countries, such as in China, lifestyle could be even less healthy although the socioeconomic status improved due to the special lifestyle transition period [40]. For older people with high SES, the influence of healthy behaviors alone in reducing mortality risk is relatively limited. Beyond lifestyle factors, other possible mediators, such as medical care, chronic stress, sleep, neighborhood condition, and epigenetic expressions [41], should be investigated.

### Strengths and Limitations of This Study

Strengths of our study included the large well-established nationwide sample, its prospective cohort study design, and a long follow-up time. The five waves of CLHLS cohort included in the present study had its own specialty. The populations were characterized by the widened SES due to the socioeconomic transformations over the past century, and thus can shed a light on the association of lifestyle factors and socioeconomic inequity in health more profoundly. We also adjusted for as many factors as possible that may be associated with SES and mortality, which may greatly reduce unmeasured residual confounding. In addition, we have conducted stratification analyses and a series of sensitivity analyses to support robustness of the present findings. These findings might fill the gap in respect to the mediating effect of healthy lifestyles in the association of socioeconomic inequity in mortality for older Chinese people.

There are several limitations in the present study. First, we have used occupation before participants’ 60s as one of the three indicators to determine personal SES, which might be problematic for older populations because of the hypothesized links between work environments and health. Besides, family income is based on self-reported data, which might not be accurate because of the sensitivity of the topic. However, collecting comprehensive and accurate data on income per se is difficult [42]. Second, this study gave equal weight for each unhealthy behaviors without considering the variation in dose, frequency or the duration (e.g., frequency and pack-years of smoking). This kind of nondifferential errors in the measurement of health behaviors might lead to biased results. Third, population SES could change over time as well, however, it is unlikely to receive adult education or have significant income fluctuation for older people. Fourth, our study cohort represents a relative disadvantaged fraction of the population, in which participants categorized as high SES only accounted for 10.8%. The current findings might be challenged in other more even or well-developed socioeconomic contexts. Contrasting the role of lifestyle factors in socioeconomic gradients in health among different countries is encouraged. Fifth, some other possible important mediators of the SES-health association, such as medical care access, were not included in the present study, and thus we cannot comment on the mediating effect of other variables. Finally, although we have considered potential confounding factors as much as possible, e.g., personal characteristics and comorbidities, residual confounding factors still possibly exist, which might reduce the strength of the results.

## Conclusion

Based on the CLHLS cohort, low SES was significantly associated with higher risk of mortality in older Chinese people, and the association was only modestly mediated by lifestyle factors. Promotion of healthy behaviors alone could only reduce a small proportion of SES-related mortality risk in older people. Therefore, other social determinants of health should be further addressed for older people. Nonetheless, for older people within different levels of SES, mortality risk showed a downward trend with increased number of healthy behaviors, especially in low and medium SES. Those results indicated the importance of lifestyle modification in reducing overall mortality for older people, especially those of low and medium SES.

## Supplementary Information

Below is the link to the electronic supplementary material.

Supplementary file1 (DOCX 43 KB)

eFigure 1. Spatial distributions of the study population. In the present study, province with the most study participants was Jiangsu (n = 3,856), followed by Guangxi (n = 3,731), Shandong (n = 3,168), Sichuan, Zhejiang, Henan, Guangdong, Hunan, Anhui, Hubei, Liaoning, Shanghai, Chongqing, Fujian, Jilin, Helongjiang, Beijing, Hainan, Jiangxi, Shaanxi, Tianjin (n = 354), Hebei (n = 309), and Shanxi (n = 304) (TIF 3876 KB)

eFigure 2. Distribution of socioeconomic status scores among the present study participants (TIF 877 KB)

Supplementary file4 (DOCX 18 KB)

Supplementary file5 (DOCX 19 KB)

Supplementary file6 (DOCX 16 KB)

Supplementary file7 (DOCX 16 KB)

Supplementary file8 (DOCX 19 KB)

Supplementary file9 (DOCX 17 KB)

Supplementary file10 (DOCX 16 KB)

Supplementary file11 (DOCX 18 KB)

Supplementary file12 (DOCX 16 KB)

## Data Availability

Researchers can download the datasets free of charge from the following websites: (1) https://opendata.pku.edu.cn; Peking University Open Access Research Database; (2) https://www.icpsr.umich.edu/icpsrweb/NACDA/series/487; National Archive of Computerized Data on Aging (NACDA) sponsored by U.S. National Institute of Aging (NIA/NIH), Inter-university Consortium for Political and Social Research (ICPSR) at University of Michigan.

## Abbreviations

SES: Socioeconomic status
CLHLS: Chinese Longitudinal Healthy Longevity Survey
ADL: Activities of daily living
IQR: Interquartile range
HR: Hazard ratio
