# Supplementary material for: Promotion of Healthy Lifestyles Alone Might Not Substantially Reduce Socioeconomic Inequity-Related Mortality Risk in Older People in China: A Prospective Cohort Study
Source: J Epidemiol Glob Health. 2023 Mar 4;13(2):322–32. doi: 10.1007/s44197-023-00095-3 (PMC10272001; doi:10.1007/s44197-023-00095-3)
Supplement: Supplementary file 1 — Supplementary file1 (DOCX 43 KB) [file 44197_2023_95_MOESM1_ESM.docx]

**eMethods**

**Assessment of socioeconomic status**

Education level, occupation, and income were used to measure socioeconomic status (SES) according to previous studies^1 2^. Because the information about income was collected since wave 2002, we included the older participants (≥ 65 years) from the 2002 survey and newly recruited participants from the follow-up surveys. As follows:

- Educational levels were coded into three categories: 0 years (no school, score = 1); 1–6 years (primary school, score = 2); and ≥ 7 years (middle school or more, score = 3).
- Because all participants in our study have retired (age ≥ 65 years), we used the primary occupation before age 60 as the occupational levels. Based on the previous study^3^, the occupation included three levels: agriculture, forestry, animal husbandry, fishery, housewife, unemployed or others (low occupational grade, score = 1); staff, service/industrial worker, self-employer or military personnel (medium occupational grade, score = 2); and professional/ technical personnel, doctors or teachers, governmental/institutional/managerial personnel (high occupational grade, score = 3).
- Based on the question “How do you rate your economic status compared with others in your local area?”, the income was bracketed into three categories: very poor or poor (poor, score = 1); fair (fair, score = 2); very rich or rich (rich, score = 3).

(Note: if the variables` answers were “do not know” or “missing”, we uniformly defined them as missing)

The SES score assigned to each participant was the sum of education level, occupation, and income scores, as previously described^3 4^.

In addition, some studies have suggested health insurance as an additional indicator of socioeconomic status^3 5^. However, health insurance in China includes multiple types, such as public free medical services, medical insurance for urban workers, collective medical insurance for urban residents, new rural cooperative medical insurance, commercial medical insurance, and others. Meanwhile, the same type of health insurance may have a different reimbursement rate in different regions, and the reimbursement rate may overlap among different types of health insurance for different diseases. Therefore, using health insurance as an additional indicator of socioeconomic status might not be practical with consideration of complexities of health insurance in China.

**Assessment of healthy lifestyles**

Since multiple lifestyle factors are interrelated and are associated with mortality and morbidity, we constructed a healthy lifestyle score including cigarette smoking, alcohol consumption, physical activity, and diet, which also coincided with recommendations from the World Health Organization (<https://apps.who.int/iris/bitstream/handle/10665/259232/WHO-NMH-NVI-17.9-eng.pdf?sequence=1&isAllowed=y>.). As follows:

- Cigarette smoking: In the CLHLS, participants were asked about their current cigarette smoking status, including yes and no. Those who did not smoke at present would be asked about the past cigarette smoking status, including yes and no. Then, smoking status was categorized into current, former and never. Although all-cause mortality may reduce to the level of never smokers with the extended time period of smoking cessation^6^, overall mortality risk remains high, in spite of no statistical significance. Combined with the study about smoking cessation in the oldest old people^7^, which showed that participants who smoked currently and those who smoked formerly had a higher risk of all-cause mortality compared to never smokers, we defined never smoking as a healthy level in the present study.
- Alcohol consumption: Participants were asked about their current and past alcohol consumption status, including yes and no. Then, alcohol consumption status was categorized into current, former and never. In addition, the information on alcohol consumption also included the type (very strong liquor, ≥ 38% alcohol; not very strong liquor, < 38% alcohol; wine, rice wine, beer, and others) and the amount per day (Liang [Chinese Unit]; 1 Liang equals to 50 g). We assumed that each participant drank one type per time, and used the aforementioned information to calculate the total amount of pure alcohol that each participant drank daily, assuming the following common alcohol content by volume (v/v) in China: very strong liquor 53%, not very strong liquor 35%, wine 14%, rice wine 18%, beer 4%, and others (average alcohol content of the aforementioned five types 25%).

For example, a participant drinks 1 Liang (50 g) of very strong liquor (v/v: 53%), and the amount of pure alcohol was calculated as:

- First, density of very strong liquor (v/v: 53%, 100 ml):

$\left( 1\frac{g}{ml}\times47 ml \left[ density and volume for pure water \right]+ 0.789\frac{g}{ml}\times53 ml \left[ density and volume for pure alcohol \right] \right)\div100 ml = 0.888\frac{g}{ml}$

- Second, volume for 50 g very strong liquor:

$50 g \div0.888\frac{g}{ml}= 56.3 ml$

- Third, amount of pure alcohol for 50 g very strong liquor:

$56.3 ml \times53\% \times0. 888\frac{g}{ml}= 26.5 g$

(Therefore, the densities were 0.926 g/ml, 0.970 g/ml, 0.962 g/ml, 0.992 g/ml, and 0.947 g/ml for not very strong liquor 35%, wine 14%, rice wine 18%, beer 4%, and others 25%, respectively.)

Chinese dietary guidelines (2022) recommends that adults should drink no more than 15 g of pure alcohol per day. On the other hand, previous studies have shown that former drinkers have a higher mortality risk compared to never drinkers^8 9^; therefore, former drinkers (> 15 g of pure alcohol per day) were excluded from the healthy group. In the present study, a healthy level was defined as never drinking or daily consumption of ≤ 15 g pure alcohol in the past or at present, i.e. no heavy alcohol consumption.

- Physical activity: According to WHO 2020 guidelines on physical activity and sedentary behavior^10^, physical activity was defined as any bodily movement produced by skeletal muscles that requires energy expenditure. Therefore, the present study relied on two survey questions asking whether the participant exercised regularly at present (yes or no), and the frequency of engagement in active leisure activities, which included raising domestic animals, taking part in some social activities, doing house work, any personal outdoor activities, and garden work in the CLHLS. Those who exercised regularly at present or participated in any active activity almost everyday were defined as a healthy level.
- Diet: According to a previous CLHLS study^11^, we calculated scores for food items, including healthful plant foods (whole grains, fruits, fresh vegetables, beans, garlic, and tea), unhealthful plant foods (refined grains, preserved vegetables, and sugar), and animal foods (eggs, fish and aquatic products, and meat). Based on the study^11^, we first defined intake frequency scores for each food item. In waves 2002 and 2005, the food groups included beans, garlic, tea, preserved vegetables, sugar, eggs, fish and aquatic products, and meat, and the intake frequency ‘almost every day’, ‘occasionally’ or ‘rarely or never’ were assigned scores of 5, 2 and 1, respectively. From wave 2008 to wave 2014, these aforementioned food groups` intake frequency ‘almost every day’, ‘≥ 1 times per week’, ‘≥ 1 times per month’, ‘occasionally’ or ‘rarely or never’ were assigned scores of 5, 4, 3, 2 and 1, respectively. For fruits and fresh vegetables from wave 2002 to wave 2014, the intake frequency was recorded as ‘almost every day’ or ‘quite often’ or ‘occasionally’ or ‘rarely or never’ and scored 5, 4, 2 and 1, respectively. For whole grains and refined grains from wave 2002 to wave 2014, the binary answers (‘as main staple food’ or not) were assigned score of 5 or 1. Therefore, a higher intake frequency score of each food group indicated more frequent consumption.

For calculating healthful diet index, we then recoded the above-mentioned scores based on the potentially divergent health effects: participants received positive scores for each food item in healthful plant food groups but reverse scores for each food item in unhealthful plant food groups and animal food groups. The healthful diet index was then calculated as the summation of scores for each food item. In the original study^11^, the top three quintiles of healthful diet index were associated with reduced risk of all-cause mortality; therefore, we also defined the top three quintiles of healthful diet index as a healthy diet in our study.

(Note: if the variables` answers were “do not know” or “missing”, we uniformly defined them as missing)

For each lifestyle factor, we assigned 1 point for a healthy level and 0 points for an unhealthy level. Thus, the healthy lifestyle score was the sum of the points and ranged between 0 and 4, with higher scores indicating healthier lifestyles, and the method has been used widely^3 4^.

**Reference**

1. Murayama H, Liang J, Bennett JM, et al. Socioeconomic status and the trajectory of body mass index among older Japanese: A nationwide cohort study of 1987–2006. *Journals of Gerontology Series B: Psychological Sciences and Social Sciences* 2016;71(2):378-88.

2. Winkleby MA, Jatulis DE, Frank E, et al. Socioeconomic status and health: how education, income, and occupation contribute to risk factors for cardiovascular disease. *American journal of public health* 1992;82(6):816-20.

3. Wu W-H, Yang L, Peng F-H, et al. Lower socioeconomic status is associated with worse outcomes in pulmonary arterial hypertension. *American journal of respiratory and critical care medicine* 2013;187(3):303-10.

4. Niu S, Zhao D, Zhu J, et al. The association between socioeconomic status of high-risk patients with coronary heart disease and the treatment rates of evidence-based medicine for coronary heart disease secondary prevention in China: Results from the Bridging the Gap on CHD Secondary Prevention in China (BRIG) Project. *American heart journal* 2009;157(4):709-15. e1.

5. Zhang Y-B, Chen C, Pan X-F, et al. Associations of healthy lifestyle and socioeconomic status with mortality and incident cardiovascular disease: two prospective cohort studies. *bmj* 2021;373

6. Yang JJ, Yu D, Shu X-O, et al. Reduction in total and major cause-specific mortality from tobacco smoking cessation: a pooled analysis of 16 population-based cohort studies in Asia. *International journal of epidemiology* 2021;50(6):2070-81.

7. Wei Y, Lv Y, Zhou J, et al. Smoking cessation in late life is associated with increased risk of all-cause mortality amongst oldest old people: a community-based prospective cohort study. *Age and Ageing* 2021;50(4):1298-305.

8. Xi B, Veeranki SP, Zhao M, et al. Relationship of alcohol consumption to all-cause, cardiovascular, and cancer-related mortality in US adults. *Journal of the American College of Cardiology* 2017;70(8):913-22.

9. Stockwell T, Zhao J, Panwar S, et al. Do “moderate” drinkers have reduced mortality risk? A systematic review and meta-analysis of alcohol consumption and all-cause mortality. *Journal of studies on alcohol and drugs* 2016;77(2):185-98.

10. Organization WH. WHO guidelines on physical activity and sedentary behaviour: web annex: evidence profiles. 2020

11. Chen H, Shen J, Xuan J, et al. Plant-based dietary patterns in relation to mortality among older adults in China. *Nature Aging* 2022;2(3):224-30.
