# Supplementary figures and images for: Promotion of Healthy Lifestyles Alone Might Not Substantially Reduce Socioeconomic Inequity-Related Mortality Risk in Older People in China: A Prospective Cohort Study
Source: J Epidemiol Glob Health. 2023 Mar 4;13(2):322–32. doi: 10.1007/s44197-023-00095-3 (PMC10272001; doi:10.1007/s44197-023-00095-3)

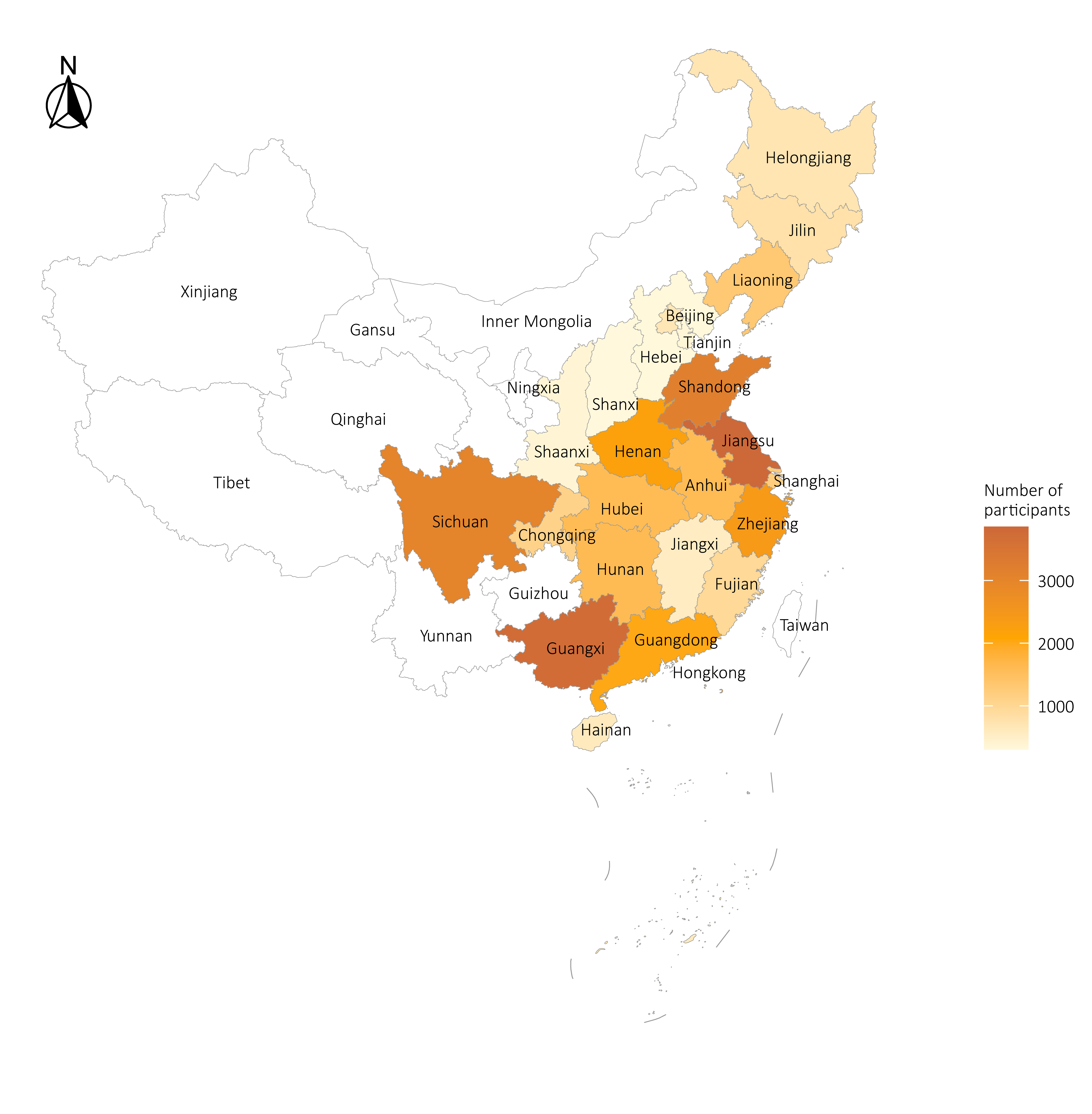

Supplement: Supplementary file 2 — eFigure 1. Spatial distributions of the study population. In the present study, province with the most study participants was Jiangsu (n = 3,856), followed by Guangxi (n = 3,731), Shandong (n = 3,168), Sichuan, Zhejiang, Henan, Guangdong, Hunan, Anhui, Hubei, Liaoning, Shanghai, Chongqing, Fujian, Jilin, Helongjiang, Beijing, Hainan, Jiangxi, Shaanxi, Tianjin (n = 354), Hebei (n = 309), and Shanxi (n = 304) (TIF 3876 KB) [file 44197_2023_95_MOESM2_ESM.tif]

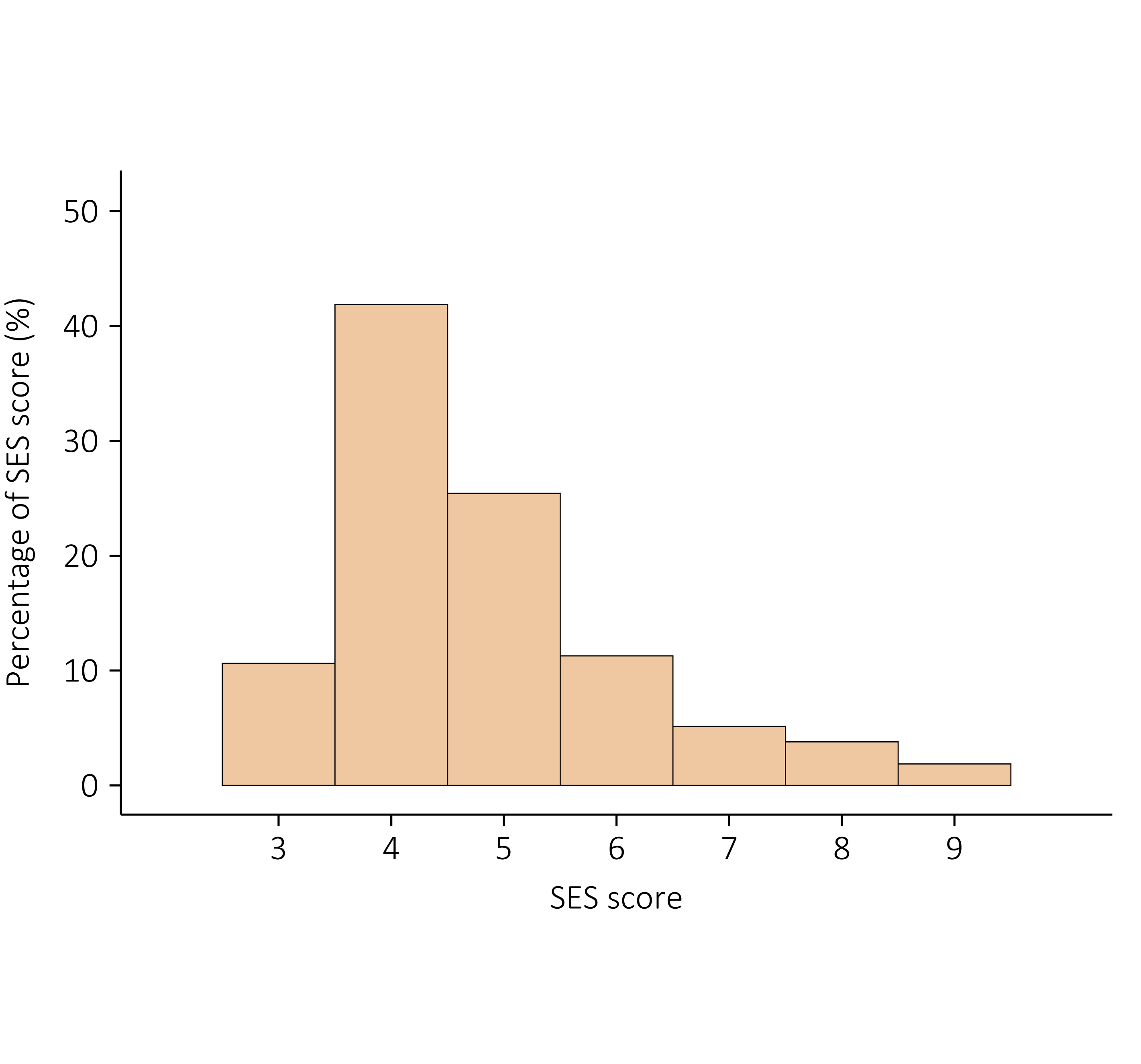

Supplement: Supplementary file 3 — eFigure 2. Distribution of socioeconomic status scores among the present study participants (TIF 877 KB) [file 44197_2023_95_MOESM3_ESM.tif]
