# Supplementary material for: Promotion of Healthy Lifestyles Alone Might Not Substantially Reduce Socioeconomic Inequity-Related Mortality Risk in Older People in China: A Prospective Cohort Study
Source: J Epidemiol Glob Health. 2023 Mar 4;13(2):322–32. doi: 10.1007/s44197-023-00095-3 (PMC10272001; doi:10.1007/s44197-023-00095-3)
Supplement: Supplementary file 4 — Supplementary file4 (DOCX 18 KB) [file 44197_2023_95_MOESM4_ESM.docx]

| eTable 1. Definitions of baseline variables in the present study ^a^ | | | | |
| --- | --- | --- | --- | --- |
| Variable | Components of variable | Questions in CLHLS questionnaire | Options in the questionnaire | Scales of reclassification  in the present study |
| Sex |  |  | • male • female | • Male • Female |
| Age |  |  |  | • Age (years) |
| Marital status |  | Current marital status? | • currently married and living with spouse • separated • divorced • widowed • never married • don't know • missing | • In marriage: currently married and living with spouse, separated • Not in marriage: divorced, widowed, never married • missing: don't know, missing |
| Residence |  | Current residence area of interviewee? | • city • town  • rural | • Urban: city, town • Rural: rural |
| Co-residence |  | Co-residence? | • with household member(s) • alone • in an institution • missing | • With household member(s) • Alone • In an institution • missing |
| Hypertension |  | Are you suffering from this disease? | • yes • no • don't know • missing | • Yes: yes • No: no • missing: don't know, missing |
| Diabetes |  | Are you suffering from this disease? | • yes • no • don't know • missing | • Yes: yes • No: no • missing: don't know, missing |
| Heart diseases |  | Are you suffering from this disease? | • yes • no • don't know • missing | • Yes: yes • No: no • missing: don't know, missing |
| Cerebrovascular diseases |  | Are you suffering from this disease? | • yes • no • don't know • missing | • Yes: yes • No: no • missing: don't know, missing |
| Respiratory diseases |  | Are you suffering from this disease? | • yes • no • don't know • missing | • Yes: yes • No: no • missing: don't know, missing |
| Cancer |  | Are you suffering from this disease? | • yes • no • don't know • missing | • Yes: yes • No: no • missing: don't know, missing |
| ADL disability | Bathing | Without assistance? | • without assistance • one part assistance • more than one part assistance • don't know • missing | • In the CLHLS survey, six items of daily self-care ability were collected from each participant based on the Katz index: dressing, bathing, transferring, toileting, continence, and eating. Each item included three answers: complete independence, partially dependence, and complete dependence. ADL disability was defined as present if participants needed any assistance in performing at least one of the six self-care activities. |
|  | Dressing | Get clothes and get completely dressed without assistance? | • without assistance • need assistance for trying shoes • assistance in getting clothes and getting dressed • missing |  |
|  | Toileting | Go to the toilet, cleans self, and arranges clothes without assistance (may use object for support such as cane, walker, or wheelchair)? | • without assistance • assistance in cleaning or arranging clothes • don't use toilet • missing |  |
|  | Transferring | Get in and out of bed as well as in and out of a chair without assistance (may use object for support such as cane or walker)? | • without assistance • with assistance • bedridden • missing |  |
|  | Continence | Has complete control of urination and bowel movement without assistance? | • without assistance • occasional accidents • incontinent • missing |  |
|  | Feeding | Feed self without assistance? | • without assistance • with some help • need feeding • missing |  |
| Self-reported health |  | How do you rate your health at present? | • very good • good • so so • bad • very bad • not able to answer • missing | • Good: very good, good • Fair: so so • Poor: bad, very bad • missing: don't know, missing |
| ^a^ About the detailed assessment of SES and healthy lifestyles could be found in eMethods.  Other detailed information about the questionnaires could be found on: https://agingcenter.duke.edu/CLHLS. Participants with missing values defined in our study were deleted in the main statistical analyses, and we performed multiple imputation for missing values as a sensitivity analysis. Abbreviations: ADL = activities of daily living, CLHLS = Chinese Longitudinal Healthy Longevity Surveys, SES = socioeconomic status. | | | | |
