# Supplementary material for: Promotion of Healthy Lifestyles Alone Might Not Substantially Reduce Socioeconomic Inequity-Related Mortality Risk in Older People in China: A Prospective Cohort Study
Source: J Epidemiol Glob Health. 2023 Mar 4;13(2):322–32. doi: 10.1007/s44197-023-00095-3 (PMC10272001; doi:10.1007/s44197-023-00095-3)
Supplement: Supplementary file 5 — Supplementary file5 (DOCX 19 KB) [file 44197_2023_95_MOESM5_ESM.docx]

| eTable 2. Baseline characteristics of participants included or excluded from analyses | | | |
| --- | --- | --- | --- |
| Variable | Included participants  (n=22093) | Excluded participants  (n=12321)^a^ | p value^b^ |
| Sex: male | 9511 (43.05%) | 4766 (38.68%) | <0.001 |
| Age (years) | 88.00 (77.00, 96.00) | 92.00 (82.00, 100.00) | <0.001 |
| Marital status |  |  | <0.001 |
| In marriage | 7291 (33.00%) | 3109 (25.28%) |  |
| Not in marriage | 14802 (67.00%) | 9188 (74.72%) |  |
| Residence |  |  | <0.001 |
| Urban | 8851 (40.06%) | 5522 (44.82%) |  |
| Rural | 13242 (59.94%) | 6799 (55.18%) |  |
| Co-residence |  |  | <0.001 |
| With family members | 18292 (82.80%) | 10127 (82.39%) |  |
| Alone | 3213 (14.54%) | 1633 (13.29%) |  |
| In an institution | 588 (2.66%) | 532 (4.33%) |  |
| Comorbidities |  |  |  |
| Hypertension | 3756 (17.00%) | 1966 (17.95%) | 0.032 |
| Diabetes | 476 (2.15%) | 308 (2.84%) | <0.001 |
| Heart diseases | 1787 (8.09%) | 1046 (9.56%) | <0.001 |
| Cerebrovascular diseases | 1070 (4.84%) | 720 (6.54%) | <0.001 |
| Respiratory diseases | 2524 (11.42%) | 1401 (12.61%) | 0.002 |
| Cancer | 92 (0.42%) | 47 (0.44%) | 0.837 |
| ADL disability | 5145 (23.29%) | 4650 (38.14%) | <0.001 |
| Self-reported health |  |  | <0.001 |
| Good | 11281 (51.06%) | 4049 (46.45%) |  |
| Fair | 7522 (34.05%) | 3225 (37.00%) |  |
| Poor | 3290 (14.89%) | 1442 (16.54%) |  |
| Education |  |  | <0.001 |
| Middle school or more | 2040 (9.23%) | 1208 (9.99%) |  |
| Primary school | 6129 (27.74%) | 2904 (24.02%) |  |
| No school | 13924 (63.02%) | 7976 (65.98%) |  |
| Occupation before 60 years |  |  | 0.002 |
| High occupational grade | 1602 (7.25%) | 803 (6.73%) |  |
| Medium occupational grade | 3432 (15.53%) | 2019 (16.92%) |  |
| Low occupational grade | 17059 (77.21%) | 9113 (76.36%) |  |
| Income |  |  | <0.001 |
| Rich | 3676 (16.64%) | 1769 (14.58%) |  |
| Fair | 14959 (67.71%) | 8194 (67.55%) |  |
| Poor | 3458 (15.65%) | 2168 (17.87%) |  |
| Never smoking | 14769 (66.85%) | 8642 (70.47%) | <0.001 |
| No heavy alcohol consumption | 16205 (73.35%) | 8946 (77.83%) | <0.001 |
| Regular physical activity | 14823 (67.09%) | 6515 (53.05%) | <0.001 |
| Healthy diet | 13587 (61.50%) | 7996 (66.54%) | <0.001 |
| Values are median (IQR) or n (%). ^a^ Each variable was shown by actual sample size. ^b^ Baseline differences were compared using the Kruskal-Wallis test for continuous variables, and the chi-square or Fisher exact tests for categorical variables.  Abbreviations: ADL = activities of daily living, IQR = inter-quartile range. | | | |
