# Supplementary material for: Promotion of Healthy Lifestyles Alone Might Not Substantially Reduce Socioeconomic Inequity-Related Mortality Risk in Older People in China: A Prospective Cohort Study
Source: J Epidemiol Glob Health. 2023 Mar 4;13(2):322–32. doi: 10.1007/s44197-023-00095-3 (PMC10272001; doi:10.1007/s44197-023-00095-3)
Supplement: Supplementary file 6 — Supplementary file6 (DOCX 16 KB) [file 44197_2023_95_MOESM6_ESM.docx]

| eTable 3. Association of each socioeconomic factor  and the score with all-cause mortality | | | |
| --- | --- | --- | --- |
| Variable | Deaths/total | Adjusted HR (95% CI) | p value |
| Education |  |  |  |
| Middle school or more | 1063/2040 | 1 [reference] |  |
| Primary school | 3955/6129 | 1.13 (1.06-1.21) | <0.001 |
| No school | 10703/13924 | 1.18 (1.10-1.27) | <0.001 |
| Occupation before 60 years |  |  |  |
| High occupational grade | 884/1602 | 1 [reference] |  |
| Medium occupational grade | 2169/3432 | 1.12 (1.03-1.21) | 0.005 |
| Low occupational grade | 12668/17059 | 1.19 (1.10-1.28) | <0.001 |
| Income |  |  |  |
| Rich | 2588/3676 | 1 [reference] |  |
| Fair | 10526/14959 | 1.02 (0.98-1.07) | 0.296 |
| Poor | 2607/3458 | 1.00 (0.95-1.06) | 0.981 |
|  |  |  |  |
| SES score |  |  |  |
| Continuous, per 1 score decrease | 15721/22093 | 1.07 (1.06-1.08) | <0.001 |
| As categorical variable |  |  |  |
| High SES | 1298/2383 | 1 [reference] | <0.001^a^ |
| Medium SES | 5514/8110 | 1.14 (1.07-1.21) |  |
| Low SES | 8909/11600 | 1.17 (1.10-1.25) |  |
| ^a^ p value for trend, obtained from Wald tests of a linear association of the score as a numeral (1-3) with the risk of all-cause mortality. All analyses were adjusted for sex, age, marital status, residence, co-residence, comorbidities, ADL disability, self-reported health, and healthy lifestyles. Abbreviations: CI = confidence interval, HR = hazard ratio, SES = socioeconomic status. | | | |
