# Supplementary material for: Promotion of Healthy Lifestyles Alone Might Not Substantially Reduce Socioeconomic Inequity-Related Mortality Risk in Older People in China: A Prospective Cohort Study
Source: J Epidemiol Glob Health. 2023 Mar 4;13(2):322–32. doi: 10.1007/s44197-023-00095-3 (PMC10272001; doi:10.1007/s44197-023-00095-3)
Supplement: Supplementary file 7 — Supplementary file7 (DOCX 16 KB) [file 44197_2023_95_MOESM7_ESM.docx]

| eTable 4. Association of each healthy lifestyle factor  and the score with all-cause mortality. | | | |
| --- | --- | --- | --- |
| Variable | Deaths/total | Adjusted HR (95% CI) | p value |
| Never smoking | 15721/22093 | 0.89 (0.85-0.92) | <0.001 |
| No heavy alcohol consumption | 15721/22093 | 0.95 (0.92-0.99) | 0.013 |
| Regular physical activity | 15721/22093 | 0.79 (0.76-0.81) | <0.001 |
| Healthy diet | 15721/22093 | 1.02 (0.99-1.06) | 0.188 |
|  |  |  |  |
| Healthy lifestyle score |  |  |  |
| Continuous, per 1 score increase | 15721/22093 | 0.92 (0.91-0.94) | <0.001 |
| As categorical variable |  |  |  |
| 0 | 289/358 | 1 [reference] | <0.001^a^ |
| 1 | 1920/2554 | 1.01 (0.89-1.14) |  |
| 2 | 4256/5701 | 0.94 (0.83-1.06) |  |
| 3 | 6137/8492 | 0.88 (0.78-0.99) |  |
| 4 | 3119/4988 | 0.77 (0.68-0.87) |  |
| ^a^ p value for trend, obtained from Wald tests of a linear association of the score as a numeral (0-4) with the risk of all-cause mortality.  All analyses were adjusted for sex, age, marital status, residence, co-residence, comorbidities, ADL disability, self-reported health, and SES. Abbreviations: CI = confidence interval, HR = hazard ratio, SES = socioeconomic status. | | | |
