# Supplementary material for: Promotion of Healthy Lifestyles Alone Might Not Substantially Reduce Socioeconomic Inequity-Related Mortality Risk in Older People in China: A Prospective Cohort Study
Source: J Epidemiol Glob Health. 2023 Mar 4;13(2):322–32. doi: 10.1007/s44197-023-00095-3 (PMC10272001; doi:10.1007/s44197-023-00095-3)
Supplement: Supplementary file 8 — Supplementary file8 (DOCX 19 KB) [file 44197_2023_95_MOESM8_ESM.docx]

| eTable 5. Mediation (by healthy lifestyles) analysis of socioeconomic status and all-cause mortality: stratification analysis | | | | | |
| --- | --- | --- | --- | --- | --- |
|  | Deaths/total | Association^a^ | | | |
|  |  | Total effect; HR (95% CI), p | Natural direct effect;  HR (95% CI), p | Natural indirect effect; HR (95% CI), p | Mediation proportion; % (95% CI), p |
| Male |  |  |  |  |  |
| High SES | 1098/1899 | 1 [Reference] |  |  |  |
| Medium SES | 3184/4586 | 1.106 (1.028-1.179), <0.001 | 1.106 (1.027-1.179), <0.001 | 1 (0.997-1.003), 0.982 | 0.1 (-4.8 to 5.0), 0.982 |
| Low SES | 2310/3026 | 1.097 (1.016-1.184), 0.012 | 1.108 (1.025-1.196), 0.006 | 0.991 (0.985-0.995), <0.001 | -10.8 (-51.1 to -3.8), 0.012 |
| Female |  |  |  |  |  |
| High SES | 200/484 | 1 [Reference] |  |  |  |
| Medium SES | 2330/3524 | 1.251 (1.092-1.436), 0.002 | 1.252 (1.094-1.431), 0.002 | 0.999 (0.991-1.006), 0.702 | -0.6 (-5.7 to 3.5), 0.700 |
| Low SES | 6599/8574 | 1.300 (1.123-1.496), 0.002 | 1.323 (1.142-1.520), <0.001 | 0.983 (0.974-0.990), <0.001 | -7.4 (-16.5 to -3.7), 0.002 |
|  |  |  |  |  |  |
| Age: < 88 years |  |  |  |  |  |
| High SES | 707/1657 | 1 [Reference] |  |  |  |
| Medium SES | 2307/4454 | 1.202 (1.108-1.312), <0.001 | 1.204 (1.109-1.311), <0.001 | 0.998 (0.993-1.003), 0.524 | -1.0 (-4.7 to 2.0), 0.524 |
| Low SES | 2696/4642 | 1.379 (1.257-1.511), <0.001 | 1.406 (1.282-1.539), <0.001 | 0.981 (0.972-0.989), <0.001 | -6.9 (-11.6 to -3.9), <0.001 |
| Age: ≥ 88 years |  |  |  |  |  |
| High SES | 591/726 | 1 [Reference] |  |  |  |
| Medium SES | 3207/3656 | 1.127 (1.034-1.230), 0.002 | 1.120 (1.028-1.226), 0.006 | 1.006 (1.000-1.014), 0.056 | 5.5 (-0.2 to 21.8), 0.058 |
| Low SES | 6213/6958 | 1.174 (1.077-1.285), <0.001 | 1.178 (1.080-1.288), <0.001 | 0.997 (0.990-1.004), 0.366 | -2.1 (-8.2 to 2.7), 0.366 |
|  |  |  |  |  |  |
| With comorbidities^b^ |  |  |  |  |  |
| High SES | 562/1085 | 1 [Reference] |  |  |  |
| Medium SES | 1844/2759 | 1.230 (1.119-1.361), <0.001 | 1.225 (1.112-1.353), <0.001 | 1.004 (0.998-1.011), 0.170 | 2.2 (-1.0 to 6.7), 0.170 |
| Low SES | 2616/3477 | 1.294 (1.173-1.430), <0.001 | 1.309 (1.185-1.447), <0.001 | 0.989 (0.981-0.996), <0.001 | -4.8 (-9.6 to -2.0), <0.001 |
| Without comorbidities^b^ |  |  |  |  |  |
| High SES | 736/1298 | 1 [Reference] |  |  |  |
| Medium SES | 3670/5351 | 1.074 (0.990-1.158), 0.088 | 1.077 (0.991-1.161), 0.078 | 0.998 (0.993-1.003), 0.398 | -2.9 (-28.9 to 12.7), 0.450 |
| Low SES | 6293/8123 | 1.089 (1.001-1.175), 0.048 | 1.103 (1.015-1.189), 0.022 | 0.987 (0.981-0.993), <0.001 | -15.7 (-82.5 to -4.3), 0.048 |
| ^a^ Natural direct effect and natural indirect effect estimated the effects of SES on mortality that did not or did act through the mediator (i.e. healthy lifestyles), respectively. Mediation proportion estimated the percent of SES effect, on the log(HR) scale, that acted through the mediator, i.e. healthy lifestyles. The results were calculated without considering exposure-mediator interaction. ^b^ If a participant was without any comorbidity as shown in Table 1, he/she was defined as "no”; otherwise, he/she was defined as "yes”. Each stratification adjusted for all factors (sex, age, marital status, residence, co-residence, comorbidities, ADL disability, and self-reported health) except the stratification factor itself. Grouping criteria of continuous variables were based on the median values. Abbreviations: ADL = activities of daily living, CI = confidence interval, HR = hazard ratio, SES = socioeconomic status. | | | | | |
