# Supplementary material for: Promotion of Healthy Lifestyles Alone Might Not Substantially Reduce Socioeconomic Inequity-Related Mortality Risk in Older People in China: A Prospective Cohort Study
Source: J Epidemiol Glob Health. 2023 Mar 4;13(2):322–32. doi: 10.1007/s44197-023-00095-3 (PMC10272001; doi:10.1007/s44197-023-00095-3)
Supplement: Supplementary file 9 — Supplementary file9 (DOCX 17 KB) [file 44197_2023_95_MOESM9_ESM.docx]

| eTable 6. Mediation (by healthy lifestyles) analysis of socioeconomic status and all-cause mortality: sensitivity analysis. | | | | | |
| --- | --- | --- | --- | --- | --- |
|  | Deaths/total | Association^a^ | | | |
|  |  | Total effect; HR (95% CI), p | Natural direct effect;  HR (95% CI), p | Natural indirect effect; HR (95% CI), p | Mediation proportion; % (95% CI), p |
| Excluding deaths within the first year | | |  |  |  |
| High SES | 1147/2232 | 1 [Reference] |  |  |  |
| Medium SES | 4745/7341 | 1.128 (1.054-1.202), 0.002 | 1.129 (1.056-1.202), 0.002 | 0.999 (0.996-1.003), 0.654 | -0.7 (-4.8 to 2.7), 0.656 |
| Low SES | 7579/10270 | 1.164 (1.084-1.239), <0.001 | 1.178 (1.097-1.254), <0.001 | 0.988 (0.983-0.992), <0.001 | -8.6 (-17.3 to -4.9), <0.001 |
|  |  |  |  |  |  |
| Considering the losses censored at the median (3.12 years) of the study | | |  |  |  |
| High SES | 1298/3103 | 1 [Reference] |  |  |  |
| Medium SES | 5514/9775 | 1.155 (1.081-1.225), <0.001 | 1.155 (1.081-1.227), <0.001 | 0.999 (0.996-1.003), 0.770 | -0.4 (-3.4 to 2.3), 0.770 |
| Low SES | 8909/13647 | 1.188 (1.117-1.267), <0.001 | 1.204 (1.133-1.281), <0.001 | 0.987 (0.982-0.991), <0.001 | -8.3 (-14.5 to -5.1), <0.001 |
| Considering the losses censored at the end (17.3 years) of the study | | |  |  |  |
| High SES | 1298/3103 | 1 [Reference] |  |  |  |
| Medium SES | 5514/9775 | 1.223 (1.145-1.299), <0.001 | 1.224 (1.145-1.301), <0.001 | 1 (0.996-1.003), 0.770 | -0.3 (-2.4 to 1.6), 0.770 |
| Low SES | 8909/13647 | 1.290 (1.205-1.382), <0.001 | 1.307 (1.223-1.400), <0.001 | 0.987 (0.982-0.991), <0.001 | -5.7 (-8.7 to -3.6), <0.001 |
|  |  |  |  |  |  |
| After multiple imputation^b^ |  |  |  |  |  |
| High SES | 1464/2605 | 1 [Reference] |  |  |  |
| Medium SES | 6584/9409 | 1.124 (1.057-1.187), <0.001 | 1.126 (1.058-1.189), <0.001 | 0.999 (0.995-1.002), 0.374 | -1.2 (-5.2 to 1.7), 0.374 |
| Low SES | 11759/14800 | 1.133 (1.065-1.197), <0.001 | 1.148 (1.08-1.212), <0.001 | 0.987 (0.982-0.991), <0.001 | -11.0 (-23.2 to -6.6), <0.001 |
| ^a^ Natural direct effect and natural indirect effect estimated the effects of SES on mortality that did not or did act through the mediator (i.e. healthy lifestyles), respectively. Mediation proportion estimated the percent of SES effect, on the log(HR) scale, that acted through the mediator, i.e. healthy lifestyles. The results were calculated without considering exposure-mediator interaction. ^b^ Calculation within 1 completed dataset. Abbreviations: ADL = activities of daily living, CI = confidence interval, HR = hazard ratio, SES = socioeconomic status. | | | | | |
