# Supplementary material for: Promotion of Healthy Lifestyles Alone Might Not Substantially Reduce Socioeconomic Inequity-Related Mortality Risk in Older People in China: A Prospective Cohort Study
Source: J Epidemiol Glob Health. 2023 Mar 4;13(2):322–32. doi: 10.1007/s44197-023-00095-3 (PMC10272001; doi:10.1007/s44197-023-00095-3)
Supplement: Supplementary file 10 — Supplementary file10 (DOCX 16 KB) [file 44197_2023_95_MOESM10_ESM.docx]

| eTable 7. Association of healthy lifestyles with all-cause mortality in the group excluding deaths within the first year: sensitivity analysis. | | | | |
| --- | --- | --- | --- | --- |
|  | No. of  healthy lifestyles | Adjusted HR (95%CI), p | p for trend^a^ | p for interaction |
| High SES |  |  |  |  |
|  | 0 | 1 [Reference] |  |  |
|  | 1 | 1.12 (0.77-1.63), 0.545 |  |  |
|  | 2 | 0.97 (0.67-1.40), 0.874 | 0.057 |  |
|  | 3 | 0.93 (0.64-1.34), 0.681 |  |  |
|  | 4 | 0.93 (0.62-1.39), 0.723 |  |  |
| Medium SES |  |  |  |  |
|  | 0 | 1 [Reference] |  |  |
|  | 1 | 1.03 (0.85-1.24), 0.765 |  |  |
|  | 2 | 0.98 (0.82-1.17), 0.814 | <0.001 | 0.772 |
|  | 3 | 0.95 (0.79-1.14), 0.564 |  |  |
|  | 4 | 0.82 (0.68-0.99), 0.041 |  |  |
| Low SES |  |  |  |  |
|  | 0 | 1 [Reference] |  |  |
|  | 1 | 1.03 (0.81-1.31), 0.804 |  |  |
|  | 2 | 0.91 (0.73-1.15), 0.444 | <0.001 |  |
|  | 3 | 0.85 (0.67-1.07), 0.161 |  |  |
|  | 4 | 0.75 (0.60-0.95), 0.017 |  |  |
| ^a^ The values were obtained from Wald tests of a linear association of the score as a numeral (0-4) with the risk of all-cause mortality. All models were adjusted for sex, age, marital status, residence, co-residence, comorbidities, ADL disability, and self-reported health. Abbreviations: CI = confidence interval, HR = hazard ratio, SES = socioeconomic status. | | | | |
