# Supplementary material for: Promotion of Healthy Lifestyles Alone Might Not Substantially Reduce Socioeconomic Inequity-Related Mortality Risk in Older People in China: A Prospective Cohort Study
Source: J Epidemiol Glob Health. 2023 Mar 4;13(2):322–32. doi: 10.1007/s44197-023-00095-3 (PMC10272001; doi:10.1007/s44197-023-00095-3)
Supplement: Supplementary file 11 — Supplementary file11 (DOCX 18 KB) [file 44197_2023_95_MOESM11_ESM.docx]

| eTable 8. Association of healthy lifestyles with all-cause mortality in considering the losses censored at two time points: median (3.12 years) and the end of follow-up (17.3 years) (sensitivity analysis) | | | | |
| --- | --- | --- | --- | --- |
|  | No. of  healthy lifestyles | Adjusted HR (95%CI), p | p for  trend^a^ | p for interaction |
| Considering the losses censored at the median of the study |  |  |  |  |
| High SES |  |  |  |  |
|  | 0 | 1 [Reference] |  |  |
|  | 1 | 1.09 (0.77-1.54), 0.623 |  |  |
|  | 2 | 1.04 (0.74-1.46), 0.812 | 0.035 |  |
|  | 3 | 0.95 (0.68-1.33), 0.772 |  |  |
|  | 4 | 0.87 (0.60-1.27), 0.477 |  |  |
| Medium SES |  |  |  |  |
|  | 0 | 1 [Reference] |  |  |
|  | 1 | 1.00 (0.84-1.19), 0.996 |  |  |
|  | 2 | 0.96 (0.82-1.14), 0.654 | <0.001 | 0.996 |
|  | 3 | 0.88 (0.74-1.03), 0.118 |  |  |
|  | 4 | 0.78 (0.65-0.93), 0.005 |  |  |
| Low SES |  |  |  |  |
|  | 0 | 1 [Reference] |  |  |
|  | 1 | 1.03 (0.84-1.28), 0.751 |  |  |
|  | 2 | 0.94 (0.77-1.15), 0.552 | <0.001 |  |
|  | 3 | 0.86 (0.71-1.06), 0.157 |  |  |
|  | 4 | 0.76 (0.61-0.93), 0.008 |  |  |
|  |  |  |  |  |
| Considering the losses censored at the end of the study |  |  |  |  |
| High SES |  |  |  |  |
|  | 0 | 1 [Reference] |  |  |
|  | 1 | 1.05 (0.75-1.48), 0.766 |  |  |
|  | 2 | 1.07 (0.77-1.50), 0.676 | 0.022 |  |
|  | 3 | 0.99 (0.71-1.39), 0.975 |  |  |
|  | 4 | 0.77 (0.53-1.12), 0.171 |  |  |
| Medium SES |  |  |  |  |
|  | 0 | 1 [Reference] |  |  |
|  | 1 | 0.94 (0.79-1.12), 0.494 |  |  |
|  | 2 | 0.94 (0.79-1.11), 0.450 | <0.001 | 0.152 |
|  | 3 | 0.81 (0.68-0.95), 0.011 |  |  |
|  | 4 | 0.72 (0.61-0.86),<0.001 |  |  |
| Low SES |  |  |  |  |
|  | 0 | 1 [Reference] |  |  |
|  | 1 | 1.15 (0.93-1.42), 0.191 |  |  |
|  | 2 | 1.07 (0.87-1.31), 0.519 | <0.001 |  |
|  | 3 | 0.95 (0.77-1.16), 0.604 |  |  |
|  | 4 | 0.88 (0.72-1.08), 0.232 |  |  |
| ^a^ The values were obtained from Wald tests of a linear association of the score as a numeral (0-4) with the risk of all-cause mortality. All models were adjusted for sex, age, marital status, residence, co-residence, comorbidities, ADL disability, and self-reported health. Abbreviations: CI = confidence interval, HR = hazard ratio, SES = socioeconomic status. | | | | |
