# Supplementary material for: Promotion of Healthy Lifestyles Alone Might Not Substantially Reduce Socioeconomic Inequity-Related Mortality Risk in Older People in China: A Prospective Cohort Study
Source: J Epidemiol Glob Health. 2023 Mar 4;13(2):322–32. doi: 10.1007/s44197-023-00095-3 (PMC10272001; doi:10.1007/s44197-023-00095-3)
Supplement: Supplementary file 12 — Supplementary file12 (DOCX 16 KB) [file 44197_2023_95_MOESM12_ESM.docx]

| eTable 9. Association of healthy lifestyles with all-cause mortality  after multiple imputation: sensitivity analysis | | | | |
| --- | --- | --- | --- | --- |
|  | No. of  healthy lifestyles | Adjusted HR (95%CI), p | p for trend^a^ | p for interaction |
| High SES |  |  |  |  |
|  | 0 | 1 [Reference] |  |  |
|  | 1 | 1.16 (0.84-1.61), 0.363 |  |  |
|  | 2 | 1.05 (0.76-1.44), 0.774 | 0.011 |  |
|  | 3 | 0.94 (0.68-1.29), 0.705 |  |  |
|  | 4 | 0.94 (0.66-1.33), 0.716 |  |  |
| Medium SES |  |  |  |  |
|  | 0 | 1 [Reference] |  |  |
|  | 1 | 1.06 (0.90-1.25), 0.456 |  |  |
|  | 2 | 1.00 (0.86-1.17), 0.989 | <0.001 | 0.817 |
|  | 3 | 0.95 (0.81-1.11), 0.540 |  |  |
|  | 4 | 0.84 (0.71-0.99), 0.043 |  |  |
| Low SES |  |  |  |  |
|  | 0 | 1 [Reference] |  |  |
|  | 1 | 0.95 (0.79-1.14), 0.585 |  |  |
|  | 2 | 0.88 (0.73-1.05), 0.145 | <0.001 |  |
|  | 3 | 0.85 (0.71-1.01), 0.070 |  |  |
|  | 4 | 0.71 (0.59-0.85), <0.001 |  |  |
| ^a^ The values were obtained from Wald tests of a linear association of the score as a numeral (0-4) with the risk of all-cause mortality. All models were adjusted for sex, age, marital status, residence, co-residence, comorbidities, ADL disability, and self-reported health. The analyses used multiple imputation by chained equations to create 10 datasets from 5 iterations (the default), of which the resultant model estimates for each were combined using Rubin’s rules. Abbreviations: CI = confidence interval, HR = hazard ratio, SES = socioeconomic status. | | | | |
|  |  |  |  |  |
